# Supplementary material for: Search for 22Na in novae supported by a novel method for measuring femtosecond nuclear lifetimes
Source: Nat Commun. 2023 Sep 5;14:4536. doi: 10.1038/s41467-023-40121-3 (PMC10480179; doi:10.1038/s41467-023-40121-3)
Supplement: Supplementary file 1 — Supplementary Information [file 41467_2023_40121_MOESM1_ESM.pdf]

Supplementary information
Search for ^{22}Na in novae supported by a novel method for measuring femtosecond nuclear lifetimes

Supplementary Discussion

Experimental perspectives

In addition to the results obtained for the astrophysical state, new experimental results were obtained for about twenty ^{23}Mg states observed in this study and will be the subject of a future publication. The present method is more universal than the conventional Doppler Shift Attenuation Method (DSAM)[1–3]. Unlike DSAM where β_{reac} is not experimentally accessed and its change in the target is a source of uncertainty, in the present method a thick target can be used since β_{reac} and β_{ems} are determined for each event. This feature explains the low sensitivity of the obtained results to the ^{3}He initial implementation profile and its evolution with time, as shown in Table 1 (main text). Indeed, measuring β_{reac}, event by event, is equivalent to measuring the reaction vertex, and therefore, to an on-line measurement of the ^{3}He profile in the target and its evolution. Moreover, if the state decays mainly by proton emission instead of γ emission, it is possible to determine its lifetime with the same method using $\alpha - p$ correlations. The protons are kinetically boosted at forward angles, as the γ rays are Doppler shifted. From this kinetic-boost, it is possible to determine the velocity β_{ems} of the nucleus at the time of proton-emission and to deduce the lifetime of the state with a femtosecond accuracy. In addition, it is possible to constrain the spin of the state using the particle-particle correlations[4], by measuring the angular distribution of the second emitted particle, proton or γ, over a wide range of angles. It might be interesting to also measure the ^{23}Mg recoil nuclei. Given that the magnetic rigidity of ^{23}Mg is very different from that of the primary beam, the background from fusion-evaporation reactions could be reduced with the measurement and identification of the recoil nuclei with a magnetic spectrometer. All these advantages, obtained or achievable with remarkable detectors like AGATA and VAMOS++, make this method a promising way to measure in the future other excited states with femtosecond lifetimes.

Supplementary Methods

Sensitivity and resolution

The lifetime of the states is the unknown parameter that is to be determined with the method presented in this work. Other parameters can also be taken as free parameters, even though they are known in principle, and can be used to verify robustness of the lifetime measurement. Several parameters, such as the center-of-mass energy of the γ-ray transition, the position of the target and the implementation depth of ^{3}He ions in targets, have been considered as free parameters in the fit. Fig. 1 shows the obtained χ^2 values of the fits as a function of the γ-ray center-of-mass energy $E_{\gamma,0}$ and the lifetime τ. The

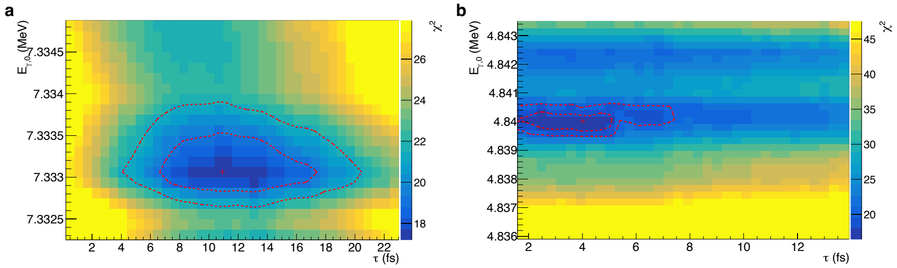

Supplementary Fig. 1 Energy versus lifetime of two excited states in ^{23}Mg. The matrices show the Pearson χ^2 test between experimental and simulated velocity-difference profiles as a function of the state lifetime τ and the γ-ray center-of-mass energy $E_{\gamma,0}$. **a** For the $E_x = 7785.0(7)$ keV state. **b** For the $E_x = 5292.0(6)$ keV state. See text for details. The dashed red lines show the limits for one and two σ.

Pearson χ^2 test, implemented in C++/ROOT[5], was used. In Fig. 1 (a), the $E_x = 7785.0(7)$ keV key state clearly shows a minimum at $\tau = 11^{+7}_{-5}$ fs. In Fig. 1 (b), the short-lived state at $E_x = 5292.0(6)$ keV, measured here with $\tau = 4^{+1}_{-3}$ fs, in good agreement with the known value $\tau = 5(2)$ fs[6], thus validating the present method of measuring very short lifetimes. The observed $\chi^2/ndf = 0.4$ (a) and 0.5 (b) indicate that errors are conservative[7], establishing the reliability of the measured lifetimes and their associated errors. In addition, our approach brings accurate values of energy of the γ-ray transitions. Here, the measured γ-ray transition energy $E_{\gamma,0} = 7333.0^{+0.5}_{-0.2}$ keV agrees with the referenced value of $E_{\gamma,0} = 7333.2(11)$ keV[6]. The Doppler shifted γ-ray transition from the key state is shown in Fig. 2. The background, from coincidences between a Compton γ ray and an α particle produced in fusion evaporation reactions with the small amount of ^{12}C and ^{16}O in the target, can be seen. This figure also highlights the advantages of this novel method, since events measured at all angles are combined into a single spectrum, i.e. the angle-integrated velocity-difference profile.

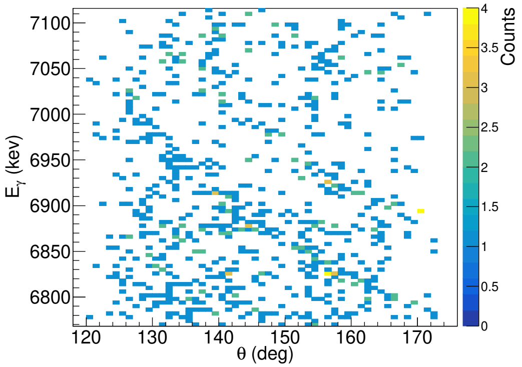

Supplementary Fig. 2 Doppler shifted 7333.2(11) keV γ-ray from the key state. This matrix with energy versus angle of the γ-rays, was measured with AGATA and was conditioned by an α particle detected in VAMOS++ in the range $7.4 < E_x < 8.2$ MeV.

Proton branching ratio

More than 20 excited states of ^{23}Mg were populated in the present work by the ^{3}He(^{24}Mg,^{4}He)^{23}Mg* reaction. The energy of the states, $E_x(^{4}\mathrm{He})$, has been determined from the energy of the ^{4}He ejectiles, measured with the VAMOS++ spectrometer. These excited states generally decay by γ-ray emission, but proton emission (^{23}Mg* $\rightarrow$^{22}Na+p) is also possible when the state is located above the proton emission threshold. SPIDER, a telescope of annular silicon detectors, was placed downstream from the target in order to measure the emitted charged particles between 10° and 25° in the laboratory frame. SPIDER is made of two silicon detectors. The first detector is 300 μm thick and segmented into 16 rings and 16 sectors. The second detector, 640 μm thick and not segmented, was used to stop the particles and to measure the residual energy E. The protons have been easily identified and selected in $\Delta E - E$ and time-of-flight matrices. The energy of the ^{23}Mg states, $E_x(p)$, has been determined from the energy of the measured protons.

Figure 3 (a) shows $E_x(p)$ versus $E_x(^{4}\mathrm{He})$ for the measured events. A background corresponding to fusion-evaporation reactions on target contaminants can be seen. The events of interest are located on the $E_x(p) = E_x(^{4}\mathrm{He})$ line, shown in Fig. 3 (a) in red, corresponding to decays to the ground state of ^{22}Na. Other decays towards excited states of ^{22}Na (^{23}Mg* $\rightarrow$^{22}Na*+p), with $E_x(^{4}\mathrm{He}) > E_x(p)$, are possible. These decays are located to the right of the line.

Figure 3 (b) shows the same matrix with the results of the Monte Carlo simulation for the astrophysical key state. A branching ratio of 100% is assumed in the simulation. All other excited states are not considered.

Figure 3 (c) shows the energies $E_x(p)$ (black points) for a portion of the events measured in the matrix around the state of interest. The background, arising from fusion-evaporation reactions, was measured during the experiment using a pure gold target and is subtracted from this spectrum. The spectrum

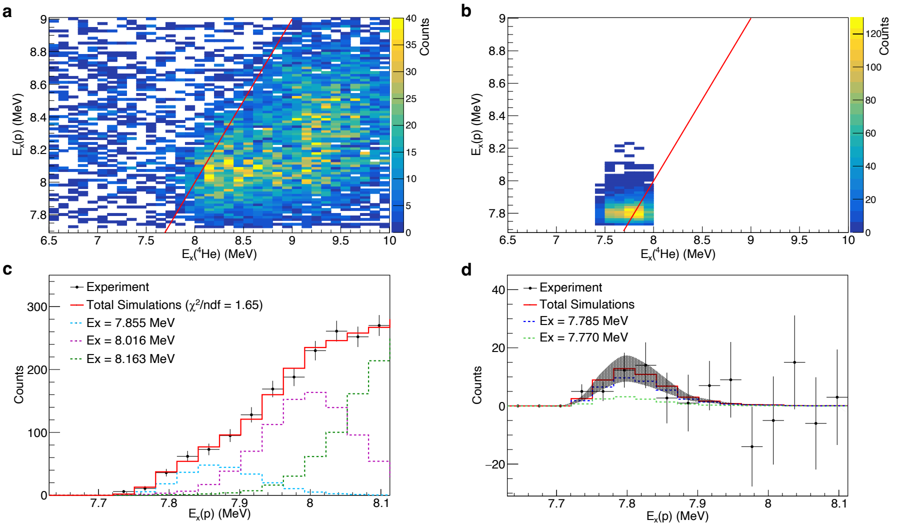

Supplementary Fig. 3 Determination of the proton branching ratio. a Excitation energy of ^{23}Mg measured using the protons $E_x(p)$ is shown against excitation energy measured using the alpha particles $E_x(^4\text{He})$. The background measured with a pure gold target has been subtracted. The events of interest are located along the $E_x(p)$=$E_x(^4\text{He})$ line (in red). **b** The corresponding Monte Carlo simulation for the key state. **c** Measured excitation energy $E_x(p)$ for the relevant part of the matrix shown in (a). The dashed and full line correspond to individual contribution of the three known states and the total, respectively. **d** The spectrum obtained after subtracting the three contributions shown in (c). See text for details. The red area corresponds to 1σ uncertainty. The vertical error bars correspond to the statistical uncertainty and the horizontal bars to the bin width.

was fitted with three peaks simulated with the Monte Carlo code. The simulation takes into account the proton detection efficiency, target thickness, angle and energy straggling effects. The energy of the known ^{23}Mg excited states have been used. On the high energy part, at least 3 peaks are needed to fit the data, corresponding to the ($\ell = 0$) states at E_x= 7.855, 8.016 and 8.163 MeV. As these states have not been seen in the γ-ray spectrum, hence it was not possible to deduce their branching ratios. However, if the observed γ-ray background is considered as an upper limit of observation, it is possible to deduce the corresponding limits (combining the measured resonance strength[8,9], spin[6], and assuming an upper value for the lifetime of 2 fs, 0.5 fs, and 0.1 fs, respectively) BR_p > 37%, > 49% and > 74% for these states, in agreement with their known values BR_p > 80%, >78% and >82%.

Figure 3 (d) shows the same spectrum after subtracting the contributions of the 3 states shown in Fig. 3 (c). Two states should be fitted at low energy. The first peak at E_x = 7768.9(15) keV is known, it has τ = 2(1) fs[10] and $\omega\gamma$ <0.5 meV[8] (and has been seen in the γ-ray spectrum). The height of the peak is therefore constrained, with BR_p < 0.2 %. Thus, only the key-state peak (in blue) is fitted to the data. The measured absolute intensity of this peak, combined with the absolute intensity of the measured γ-ray transition,

allowed the determination of the proton branching ratio. The obtained value, $BR_p = 0.68(17)\%$, is in good agreement with the latest published value, $BR_p = 0.65(8)\%$[11].

Numerical simulations

A new C++/ROOT[5] simulation code has been implemented in the present study including the complete experimental set-up. The code[12], called `EVASIONS` for *Experimental VAMOS++ AGATA SPIDER Implementation On Nuclear Spectroscopy*, follows a Monte Carlo approach to simulate the population and decay of excited ^{23}Mg states. It can be easily adapted to any other case. Kinematics is governed by energy and momentum conservation in a two-body reaction.

The measured beam characteristics and the ^{3}He implementation profile, calculated with the `SRIM` code[13], were taken into account. The slowing down of the ions inside the target is calculated using the stopping powers from `SRIM`, and γ-ray emission follows the exponential decay law. The observables associated with the particles, α, protons and the γ rays, are constructed using the experimental instrument response functions, measured prior and during the experiment. All particle emissions are isotropic. Note that protons emitted from the key state have $\ell = 0$[4]. The simulations generate different kinds of spectra, from velocity profiles at the time of emission to energy spectrum of protons emitted by ^{23}Mg.

Certain parameters, such as energy losses in the target, are not known precisely. A systematic uncertainty on these parameters has thus been added. For the states at $E_x = 5292.0(6)$ keV and $E_x = 3796.0(1.2)$ keV, the lifetimes measured in this work, 4^{+1}_{-3} fs and 40^{+6}_{-7} fs respectively, are in agreement with those reported in literature of 5(2) fs[6] and 41(6) fs[6], thus validating the simulation and analysis tools.

Supplementary Note

Thermonuclear reaction rate

Prior to the present work, the different ^{22}Na$(p,\gamma)^{23}$Mg reaction rates available led to a factor of 10 uncertainty[14] in the estimate of the amount of ^{22}Na ejected during a nova explosion. The rate was re-evaluated with the new measured lifetimes and spectroscopic data, by using the Monte Carlo statistical approach of Ref.[15]. The new rate, shown with the red curve in Fig. 4, is compared to earlier estimates. The colored bands correspond to 1σ uncertainty. The values of the new rate are also given along temperature in Table 1. The recommended rate, based on the present work, is up to ten times (three times) smaller than the rate of Ref.[8,10] (Ref.[9]), and the uncertainties are now of 10-40 % at the nova peak temperatures. These uncertainties are the most important for the production of ^{22}Na in novae, the uncertainties at lower and higher temperatures are also mentioned in Table 1.

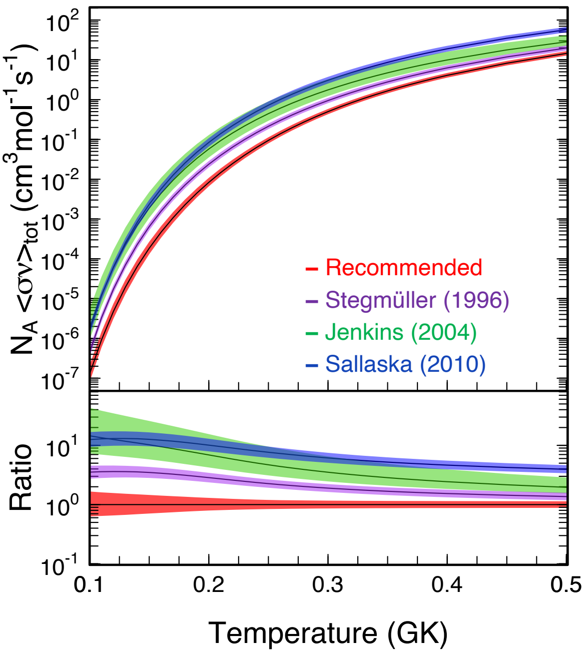

Supplementary Fig. 4 Thermonuclear reaction rate of ^{22}Na$(p,\gamma)^{23}$Mg. (Top) The recommended rate, determined with the new results obtained on the $E_x = 7785.0(7)$ keV excited state, is shown with the red curve, and compared with the former rates of Ref. [8] (blue curve), Ref. [10] (green curve), and Ref. [9] (purple curve). (Bottom) The ratios of rates, relative to the recommended rate, are also shown. The colored bands correspond to 1σ uncertainty.

The uncertainties in the new recommended rate, based on this work, result in an uncertainty of a factor of 1.4 in the estimated yield of ^{22}Na, when a representative case (i.e., Model 125, Table 2 in the main text) is adopted. For comparison, the uncertainties of previous yield estimations were of a factor of 4[11] and 3.25[10].

Supplementary Table 1 Low, recommended and high thermonuclear rates for the ^{22}Na(p,γ)^{23}Mg reaction in units of cm^3 mol^{-1} s^{-1} as a function of temperature. Recommended and 1σ limits rates have been obtained according to the approach of Ref. [15].

T (GK)	Low	Recommended	High
0.010	6.78×10^{-94}	8.18×10^{-93}	5.01×10^{-92}
0.050	1.73×10^{-17}	3.66×10^{-17}	9.91×10^{-17}
0.080	3.24×10^{-10}	5.39×10^{-10}	9.90×10^{-10}
0.100	8.08×10^{-8}	1.28×10^{-7}	2.13×10^{-7}
0.120	3.15×10^{-6}	4.80×10^{-6}	7.52×10^{-6}
0.140	4.45×10^{-5}	6.46×10^{-5}	9.63×10^{-5}
0.160	3.40×10^{-4}	4.69×10^{-4}	6.66×10^{-4}
0.180	1.73×10^{-3}	2.28×10^{-3}	3.09×10^{-3}
0.200	6.63×10^{-3}	8.35×10^{-3}	1.08×10^{-2}
0.220	2.03×10^{-2}	2.48×10^{-2}	3.10×10^{-2}
0.240	5.23×10^{-2}	6.28×10^{-2}	7.61×10^{-2}
0.260	1.17×10^{-1}	1.39×10^{-1}	1.65×10^{-1}
0.280	2.35×10^{-1}	2.75×10^{-1}	3.24×10^{-1}
0.300	4.30×10^{-1}	5.00×10^{-1}	5.83×10^{-1}
0.320	7.30×10^{-1}	8.44×10^{-1}	9.79×10^{-1}
0.340	1.17	1.34	1.55
0.360	1.77	2.03	2.33
0.380	2.56	2.93	3.36
0.400	3.57	4.08	4.68
0.450	7.23	8.23	9.40
0.500	1.28×10	1.45×10	1.65×10
0.600	3.08×10	3.47×10	3.91×10
0.800	1.04×10^{2}	1.16×10^{2}	1.30×10^{2}
1.000	2.56×10^{2}	2.86×10^{2}	3.17×10^{2}
2.000	2.31×10^{3}	2.91×10^{3}	3.09×10^{3}
4.000	5.64×10^{3}	7.59×10^{3}	7.92×10^{3}
6.000	6.02×10^{3}	8.23×10^{3}	8.56×10^{3}
8.000	5.50×10^{3}	7.56×10^{3}	7.86×10^{3}
10.000	4.83×10^{3}	6.67×10^{3}	6.93×10^{3}

Supplementary References

1. Stahl, C., Leske, J., Lettmann, M. & Pietralla, N. Apcad—analysis program for the continuous-angle dsam. *Comput. Phys. Commun.* **214**, 174–198 (2017). https://doi.org/https://doi.org/10.1016/j.cpc.2017.01.009 .

2. Nolan, P. J. & Sharpey-Schafer, J. F. The measurement of the lifetimes of excited nuclear states. *Reports on Progress in Physics* **42** (1) (1979). https://doi.org/10.1088/0034-4885/42/1/001 .

3. Schwarzschild, A. Z. & Warburton, E. K. The measurement of short nuclear lifetimes. *Annual Review of Nuclear Science* **18** (1), 265–290 (1968). https://doi.org/10.1146/annurev.ns.18.120168.001405 .

4. Pronko, J. & Lindgren, R. Angular correlations of sequential particle decay for aligned nuclei. *Nuclear Instruments and Methods* **98** (3), 445–449

(1972). https://doi.org/10.1016/0029-554X(72)90226-1 .

5. Brun, R. & Rademakers, F. ROOT — An object oriented data analysis framework. *Nucl. Instrum. Methods Phys. Res. Sect. A: Accel. Spectrom. Detect. Associated Equip.* **389** (1), 81–86 (1997). https://doi.org/10.1016/S0168-9002(97)00048-X .

6. Shamsuzzoha Basunia, M. & Chakraborty, A. Nuclear data sheets for A=23. *Nucl. Data Sheets* **171**, 1–252 (2021). https://doi.org/10.1016/j.nds.2020.12.001 .

7. Workman, R. L. *et al. Particle Data Group. Review of Particle Physics.* (PTEPr, 2022).

8. Sallaska, A. L. *et al.* Direct Measurements of ^{22}Na$(p, \gamma)^{23}$Mg Resonances and Consequences for ^{22}Na Production in Classical Novae. *Phy. Rev. Lett.* **105**, 152501 (2010). https://doi.org/10.1103/PhysRevLett.105.152501 .

9. Stegmüller, F. *et al.* ^{22}Na(p,γ)^{23}Mg resonant reaction at low energies. *Nucl. Phy. A.* **601**, 168–180 (1996). https://doi.org/10.1016/0375-9474(96)00084-X .

10. Jenkins, D. G. *et al.* Reevaluation of the ^{22}Na(p, γ) Reaction Rate: Implications for the Detection of ^{22}Na Gamma Rays from Novae. *Phy. Rev. Lett.* **92**, 031101 (2004). https://doi.org/10.1103/PhysRevLett.92.031101 .

11. Friedman, M. *et al.* Low-energy ^{23}Al β-delayed proton decay and ^{22}Na destruction in novae. *Phys. Rev. C* **101**, 052802 (2020). https://doi.org/10.1103/PhysRevC.101.052802 .

12. Fougères, C. *et al.* Search for ^{22}Na in novae supported by a novel method for measuring femtosecond nuclear lifetimes (2023). https://doi.org/10.5281/zenodo.8044973 .

13. Ziegler, J. F., Biersack, J. P. & Ziegler, M. D. SRIM - The Stopping and Range of Ions in Matter. *(SRIM Co., United States of America)* **6th ed.** (2013) .

14. Fougères, C., de Oliveira Santos, F., Smirnova, N. A. & Michelagnoli, C. Understanding the cosmic abundance of 22Na: Lifetime measurements in 23Mg. *EPJ Web Conf.* **279**, 09001 (2023). https://doi.org/10.1051/epjconf/202327909001 .

15. Longland, R. *et al.* Charged-particle thermonuclear reaction rates: I. Monte Carlo method and statistical distributions. *Nuc. Phys. A* **841**, 1–30 (2010). https://doi.org/10.1016/j.nuclphysa.2010.04.008 .
